# Recent Formulation Advances and Therapeutic Usefulness of Orally Disintegrating Tablets (ODTs)

**DOI:** 10.3390/pharmacy8040186

**Published:** 2020-10-10

**Authors:** Maimoona Chinwala

**Affiliations:** Department of Pharmaceutical, Social & Administrative Sciences, D’Youville College School of Pharmacy, Buffalo, NY 14201, USA; maimoonachinwala@gmail.com; Tel.: +1-134-7239-3480

**Keywords:** orally disintegrating tablets, formulation, pediatric, geriatric, disintegration, taste-masking, patient compliance, therapeutics

## Abstract

This review highlights the recent formulation advances (different methods of preparation involving various novel approaches) that have been advancing the use of ODT as a popular dosage form. Furthermore, the important characteristics of ODTs that are required for patient compliance and appropriate therapeutic benefit are discussed. In addition to conventional ODTs, ODTs formulated for controlled release of pharmaceuticals and taste masking are also discussed.

## 1. Introduction

Orally disintegrating or orally dispersible tablets (ODTs) are dosage forms that disintegrate or dissolve rapidly on contact with saliva. Certain ODTs that are formulated for sustained or controlled release have the ODT attribute of rapid disintegration but do not always undergo rapid dissolution. The Center for Drug Evaluation and Research (CDER), a part of the US Food and Drug Administration (FDA), has defined an ODT as a “a solid dosage form containing medicinal substances which disintegrates rapidly, usually within a matter of seconds, when placed upon the tongue”. Two key characteristics that a dosage form labelled as an ODT must possess is a rapid disintegration time of 30 s or less, and a tablet weight of 500 mg or less [1].

In the guidance for industry document “Size, Shape, and Other Physical Attributed of Generic Tablets and Capsules” published by CDER, US FDA, it is recommended that drug manufacturers develop quality target product profiles (QTPPs) for drug candidates [2]. For ODTs, parameters such as disintegration time and tablet size are key components of QTPPs. ODTs are also different from chewable tablets in that they eliminate the need for chewing or drinking liquids. Liquefaction of the ODT occurs on the tongue, followed by the patient swallowing the liquid. Drug release from an ODT is composed of a sequence of events or processes which include initial tablet disintegration, followed by drug dissolution and subsequent drug absorption [3]. Also, unlike effervescent tablets they do not need require the extra step by the patient or the care giver to be dropped into a glass of water prior to administration. Sublingual tablets (placed under the tongue) and buccal tablets (placed in the side of the cheek or high up between the inside and of the upper lip and gum) [4] are also sometimes classified as ODTs.

ODTs, by virtue of their unique characteristics, lead to better patient compliance, especially for pediatric and geriatric patients who often experience difficulty swallowing. Dysphagia (difficulty swallowing) is estimated to afflict approximately six-million adults with 38% suffering from it for their entire lifetime [5]. Successful patient compliance can also be achieved for patients with psychiatric disorders. A study conducted examined the feasibility of formulating quetiapine (short acting atypical antipsychotic) as a sustained release and taste masked ODT based on solid lipid micro-pellets [6]. Alyami et al. have reported that among health care practitioners, ODTs are the second most popular choice for pediatric patients, with the first being liquids. They also report that 63% of practitioners are in favor of substituting many liquid formulations with a suitable ODT [7].

Alavert™ Loratadine ODT (CIMA/Wyeth Consumer Health, Madison, NJ, USA), Benadryl^®^ Fastmelt™ (Yamanouchi/Pfizer, Morris Plains, NJ, USA), Claritin^®^ RediTabs^®^ (R.P.Scherer/Schering-Plough, Kenilworth, NJ, USA), Tempra^®^ FirsTabs (CIMA/Mead Johnson, Chicago, IL, USA), Excedrin^®^ QuickTabs™ (Ethypharm/BMS, Philadelphia, PA, USA) Maxalt^®^ MLT (R.P.Scherer/Merck, Kenilworth, NJ, USA), NuLev™ (CIMA/Schwarz Pharma, Milwaukee, WI, USA), Remeron^®^ SolTabs™ (CIMA/Organon, Oss, Netherlands), Triaminic^®^ SoftChews^®^ (CIMA/Novartis Consumer Health, Basel, Switzerland), Zofran ODT^®^ (R.P.Scherer/Glaxo SmithKline, Philadelphia, PA, USA), Zomig ZMT^®^ and Rapimelt (CIMA/Astra Zeneca, Wilmington, DE, USA) and Zyprexa^®^ Zydis^®^ (R.P.Scherer/Eli Lilly, Indianapolis, IN, USA) are examples of some well-known commercial ODT formulations [8].

The advantages that ODTs offer for patient compliance were further illustrated in two recent reports [9,10]. The subdivision of various commercial ODTs was evaluated and it was demonstrated that upon exposure of half of the ODT to ambient humidity, there was rapid water absorption (due to the presence of highly hygroscopic components) followed by hydrolysis. Consequently, the authors recommend that ODTs can be subdivided for dose adjustment purposes; however, one-half of the tablet would have to be discarded. Furthermore, it has been suggested manufacturing method influences the suitability of ODTs for subdivision. ODTs containing granules in a powdered matrix structure showed poor subdivision performance whereas in comparison, tablets obtained by freeze-drying or direct compression of powder could be subdivided for immediate use.

As stated above, there are many commercially manufactured ODT dosage forms available on the market. Additionally, ODTs also lend themselves well to being extemporaneously compounded by the compounding pharmacist. Compounding suppliers such as PCCA, Medisca, etc., are able to provide appropriate tablet molds to compound molded ODTs and pellet presses to compound compressed ODTs. Single punch tableting machines can also be used to extemporaneously compound tablets. It has been reported that, based on the currently available scientific evidence and the regulatory guidance, orally disintegrating dosage forms offer an innovative solution for pediatric drug delivery [11].

## 2. Preparation of ODTs for Immediate Release Applications

The two most common and widely used methods for formulation and preparation of ODTs are the direct compression and the fusion methods or sublimation. The current literature on ODTs reports various other methods, such as freeze-dried or spray-dried particles or granules that are further incorporated into tablets, microwave-assisted treatments, etc. Some studies employ solid dispersions of drugs to be formulated into ODTs. Also, many formulation studies of ODTs widely report the use of superdisintegrants. Current findings and developments are reviewed in the following paragraphs.

Successfully formulated ODTs of furosemide prepared by direct compression method and consisting of furosemide, microcrystalline cellulose (MCC), low-substituted hydroxypropylcellulose LH-11 (L-HPC), aspartame and sodium stearyl fumarate has been reported by Gulsun et al. [12]. ODTs of loratadine were prepared using Parteck ODT and Ludiflash, both of which are commercially available tableting excipients based on co-processed mannitol. Various superdisintegrants (AcDiSol, Kollidon CL-F and Kollidon CL-SF) were also added and the direct compression method was used. The authors report that all the ODTs were characterized by acceptable friability (<1%), and the reported disintegration time was below 30 s in formulations with crospovidones as disintegrant [13]. In a study conducted to evaluate coprocessed disintegrants produced from tapioca starch and mannitol in a paracetamol ODT, it was found that the coprocessing method and disintegrant incorporation influenced drug release and produced tablets higher tensile strength and consequently lower friability [14]. ODTs of atenolol and atorvastatin combination were formulated using superdisintegrants croscarmellose sodium and Kyron-T134. Among the three different techniques used, which included direct compression, effervescent and sublimation, the ODT formulation containing Kyron-T134 (6%) and croscarmellose sodium (2%) was concluded to be the best along with sublimation method as most optimum method of preparation used [15]. ODTs containing drug combinations such as levodopa/benzylhydrazine orally disintegrating tablets (L/B ODTs) prepared using direct compression have been studied. The authors reported that considerably shorter disintegration time and faster dissolution profile were obtained under the optimum formulation (optimized using response surface methodology) with microcrystalline cellulose 25.7%, cross-polyvinylpyrrolidone 6.22% and sodium carboxymethyl starch 5.36%. Thickness, friability, hardness, and wetting time were 2.8 ± 0.05 mm, 0.46 ± 0.21%, 5.42 ± 1.1 kp and 31.2 ± 2.1 s, respectively. Furthermore, the volunteers reported that the ODTs had good palatability [16]. ODTs for enalapril maleate were prepared by direct compression of enalapril with various superdisintegrants. The antihypertensive effect of the optimized ODTs was evaluated in hypertensive rats and compared with a commercial formulation. On comparison, it was found that reduction of blood pressure was obtained in 1 h with the prepared ODT as compared to 4 h for the commercial formulation [17].

Dehghani et al. conducted a study to design, optimize and evaluate an ODT of the drug meloxicam using a menthol-based solid dispersion of the drug. The authors reported that an optimized ODT exhibited hardness (48 ± 4.3 N) and friability (0.81 ± 0.07%) and a disintegration time of 19 ± 2 s of which 77.8 ± 5.1% of drug was released within 30 min [18]. As a follow-up to a previous study where it was discovered that adhesive aggregates were formed when levofloxacin hydrate tablets and lansoprazole ODTs were suspended in water, the authors conducted another study to identify the causes of the aggregate formation. When the excipients polysorbate 80 and methacrylic acid copolymer LD were added to levofloxacin (zwitterionic), only methacrylic acid copolymer LD induced aggregate formation. After examining several zwitterionic quinolone antimicrobial drugs in the study, the authors reported that that electrostatic interaction between zwitterionic ingredients and methacrylic acid copolymer LD can result in aggregate formation under conditions where the drug and methacrylic acid copolymer LD are both sufficiently soluble [19]. Researchers have prepared amlodipine novel coated dextrin microcapsules by spray drying and incorporated them into ODTs. These microcapsules were coated and then both coated and uncoated microcapsules were tableted. Unlike the uncoated microcapsules, the tableted coated microcapsules remained intact without rupture during tableting. The authors concluded that the dextrin microcapsules coated using Eudragit^®^ EPO (Evonik Health Care, Birmingham, AL, USA) be used for an amlodipine ODT formulation [20]. In a study with the drug atorvastatin calcium, an optimized complex of the drug was prepared and incorporated into ODTs and the excipients nicotinamide, Plasdone^®^ (Ashland, Covington, KY, USA).and sodium dodecyl sulphate were found to be the most favorable excipients. The authors reported that significantly higher rates and extent of dissolution were observed from the complex and tablets compared to dissolution of pure drug [21].

Many studies report using microwave treatment to formulate ODTs. In one such study conducted, the authors reported that that microwave treatment did not promote the degradation or affect the release of famotidine ODTs and acetaminophen ODTs, while dissolution of ODTs containing ibuprofen was delayed by microwave treatment. The conclusion presented by the authors was that the microwave method would be applicable to the preparation of ODTs containing active pharmaceutical ingredients with melting points higher than 110 degrees centigrade [22]. Tanaka et al. used a microwave treatment process to prepare ODTs containing powdered tea leaves containing anti-inflammatory compounds such as chafuroside A (CFA) and chafuroside B (CFB). It was observed that the disintegration time of these tablets was considerably improved (less than 20 s) compared with the microwave-untreated tablets, and there were 7- and 11-fold increases in their CFA and CFB contents. Hardness and friability characteristics of the microwave treated tablets were found to be acceptable [23]. Lamotrigine ODTs were formulated using microwave-assisted development of ODTs based on simple direct compression tableting technology. The study confirmed the stability of lamotrigine when subject to microwave irradiation. Complexation using beta cyclodextrin resulted in rapid dissolution. In this study, a simple step of humidification enabled microwave-assisted development of ODTs by direct compression [24].

Several studies have used novel technology and approaches in the formulation of ODTs. Different nanocrystal ODTs of piroxicam were prepared to enhance piroxicam dissolution rate and saturation solubility. It was found that all piroxicam nanocrystal ODT formulations showed a higher drug dissolution rate than coarse piroxicam ODT [25]. Okuda et al. developed RACTAB^(R)^ (Towa, Osaka, Japan) technology ODTs of tamsulosin hydrochloride using enteric coated particles with favorable ODT friability (<0.5%) and hardness (>50 N) [26]. In yet another novel approach used in the formulation of ODTs, diphenhydramine ODT was developed using an integrated crystal and particle engineering approach with favorable disintegration and palatability characteristics [27]. Various ratios of banana extract, dibasic calcium phosphate and microcrystalline cellulose used in a study to prepare ODTs of banana extract by combining both natural and synthetic excipients [28]. One of the more recent applications of 3D printing has been in fabrication of ODTs. Ondansetron ODTs containing drug-cyclodextrin complexes and mannitol were prepared using selective laser sintering 3D printing, and were found to exhibit disintegration and drug release characteristics similar to a commercial ODT Vonau^®^ Flash (Biolab Pharma, São Paulo, Brazil). Since 3D printing technology itself has the advantage of lending itself to making patient customized dosage forms, manufacture of dosage forms such as ODTs by this technology further enhances its value [29]. Researchers have also attempted to use some naturally occurring materials such as Occimum gratissimum seeds (OGS) which was modified by different modification processes and used as a disintegrant in formulating ODTs by the wet granulation method [30]. Orally disintegrating tablets (ODTs) produced by lyophilization have been studied and described to have a unique porous structure which favors their orodispersible characteristics [31].

## 3. ODTs Formulated for Sustained or Controlled Release Applications

A vast majority of drugs formulated as ODTs are immediate release formulations. However, there are a few commercially available products as well and studies in literature that have examined ODT formulations for their potential to sustain or control drug release.

Amphetamine extended-release orally disintegrating tablets (Adzenys XR-ODT) is a commercially available formulation. In a study conducted by Stark et al., AMP (Amphetamine) XR-ODT was found to be well tolerated and demonstrated a pharmacokinetic profile consistent with once-daily dosing in children with attention-deficit/hyperactivity disorder (ADHD) [32]. Another such commercially available formulation is methylphenidate extended-release orally disintegrating tablets (MPH XR-ODTs). In this ODT was found to be efficacious and well tolerated in school-age children with attention-deficit/hyperactivity disorder (ADHD) in a laboratory classroom setting [33]. Elwerfalli et al. prepared nano-engineered chitosan particles to sustain the release of promethazine from a sustained release ODT. Polymers such as polyvinylpyrolidine (PVP), polyethylene glycol (PEG) and polyethylene co-acrylic acid (PEAA) were used in the fabrication of the nanoparticles which were then dispersed into the tablet matrix. Differential scanning calorimeter studies indicated that promethazine was solubilized within the nanoparticles. The ODTs exhibited friability less than 1% and had tensile strength above 2.5 N/mm. Furthermore, the authors reported that the tablets disintegrated within 40 s at the same time sustaining the drug release over 24 h [34]. As per Elwerfalli technologies such as polymer coated nanoparticles, stimuli-responsive polymers and ion-exchange resins can be used to produce robust, sustained release orally disintegrating tablets [35].

Dexlansoprazole (Dexilant^®^, Takeda, Lexington, MA, USA), which is the R-enantiomer of lansoprazole is the only dual delayed-release formulation of a proton pump inhibitor (PPI) that is commercially available. This capsule formulation contains two different sets of enteric-coated granules [36]. Dual delayed release dexlansoprazole is approved for use in adults as a 30 mg orally disintegrating tablet (ODT) or as 30 mg and 60 mg capsules. In a study comparing the biovailability of dexlansoprazole administered as two 30 mg orally disintegrating tablets or one 60 mg capsule, it was observed that the AUC was 25% lower with the ODT formulation, and similar Cmax values were seen. Additionally, 24 h intragastric pH control after administration was found to be equivalent to one dexlansoprazole 60 mg capsule. This study was a randomized, phase I, open-label, single-center, multiple-dose, two-period crossover study [37]. Oldfield et al. commented on the proven efficacy of dexlansoprazole in the healing and maintenance of erosive esophagitis and also for symptomatic non-erosive GERD. The authors conclude that long-term, dexlansoprazole will most benefit patients who fail other acid suppressive therapy; however, it proves to be a more expensive option [38].

Cho et al. developed sustained release microparticles containing tamsulosin HCl for use as an ODT formulation. A melt-adsorption method was used wherein a lipid and ethylcellulose suspension (Surelease^®^) was applied to retard drug release, and magnesium aluminometasilicate (Neusilin^®^, Fuji Chemical Industries Co.,Ltd.,Toyama, Japan) was used as adsorbent. Beeswax was selected as the lipid component due to its high mechanical strength. The researchers found that a Surelease^®^ (Colorcon Inc. Harleysville, PA, USA)-to-beeswax ratio of 1:50 resulted in the desired particle size distribution and low burst release. Furthermore, the ODT containing optimized microparticles had acceptable tablet hardness and rapid disintegration [39]. One study aimed to optimize process parameters and formulation to manufacture controlled release ODTs containing melt adsorption-particles. Melt adsorption particles containing Neusilin US2 as the adsorbent were prepared by using various waxes. The authors ran a multiple regression analysis which revealed that increasing the granulation time led to sustained release of the drug and that a high compression force crushed the coated granules which impaired sustained drug release [40].

## 4. Taste Masking in ODTs

Taste masking is an important step in the formulation of most if not all ODTs. Since ODTs are designed to rapidly disintegrate in saliva, bitter, objectionable-tasting drugs may in themselves pose a compliance issue; palatability therefore has a major role in these types of formulations. Taste masking in ODTs is accomplished using a variety of approaches. Physical taste masking involves using flavors and sweeteners in the formulation. Other approaches include encapsulating drugs with different polymeric materials to prepare microparticles which are then compressed or molded into ODTs. Some other formulation approaches use formation of a complex between the drugs and other polymers or ion-exchange resins to achieve taste masking. Below are discussed and summarized recent particular studies of ODTs where taste masking was the major objective of the study.

The taste masking of mefenamic acid was achieved by researchers using Eudragit^®^ E PO in different ratios via hot melt extrusion, solid dispersion technology. Mefenamic acid was found to be converted to an amorphous phase (hydrogen bonding occurred between the drug and carrier) in all of the solid extrudates; selected extrudates which were then formulated into ODTs had the desired taste-masking effect [41]. Bitterness of an antihistamine drug, chlorpheniramine maleate, was masked by encapsulation of the drug into Eudragit^®^ EPO microparticles using spray drying. These microparticles were then subsequently incorporated into ODTs which was found to cause a significant reduction in drug bitterness. Eudragit^®^ E PO is commonly used to achieve taste masking without affecting drug release in immediate release formulations [42]. Microparticles of the bitter tasting drug prednisolone were prepared with Eudragit^®^ EPO or E 100 using spray-drying technique. Subsequently, tablets containing microparticles, commercially available ODT excipient Pharmaburst^®^ (SPI Pharma, Wilmington, DE, USA) and lubricant were directly compressed with single-punch tablet press into ODTs. Good taste masking properties were achieved with the Eudragit E 100 polymer [43]. Clindamycin hydrochloride was taste masked by coating the drug onto microcrystalline cellulose beads followed by the addition of a taste-masking layer of Eudragit^®^ EPO. This is an example of a study where the efficiency of the taste masking of the ODTs was determined using potency and drug release analysis. The authors concluded that suitable patient compliant clindamycin ODTs can be obtained by varying the levels of the taste making polymers and other excipients in the formulation [44].

Ogata et al. studied several crystalline complexes of the drug propiverine hydrochloride (used in the treatment of overactive bladders) and concluded that the propiverine salicylic acid crystalline complex was significantly less bitter than propiverine hydrochloride and the complex formation also increased the aqueous solubility of the drug. A taste sensor was used by the researchers for this study [45]. In another study, with the same drug, propiverine-loaded masking particles were formulated with different amounts of gastric-soluble coatings as physical masking. Furthermore, the propiverine-loaded masking particles were mixed with excipients such as Ludiflash^®^ (BASF Coporation, Florham Park, NJ, USA), crospovidone, and magnesium stearate and incorporated into ODTs. A few of the ODTs also prepared with the particles were organoleptically taste masked with the addition of L-menthol, aspartame, thaumatin, and cinnamon. After testing these ODTs in healthy human volunteers, the visual analog scales (VAS) scores of bitterness, numbness, and overall palatability were found to improve with organoleptic masking; however, it was concluded that the combined use of physical and organoleptic masking was useful for improving palatability of ODTs [46]. A unique kind of a study evaluated the palatability of orally disintegrating tablets containing core granules with different particle sizes, coating, and types of materials using visual analog scales. It was observed that rough mouth feel was found to be more significant with core granules having particle sizes ≥ 200 µm and reduced rough mouth feel was observed with core granules composed of water-soluble additives [47].

An ODT formulation of donepezil hydrochloride prepared using wet granulation and excipients such as Crospovidone XL-10 and three different grades of lactose was evaluated for palatability in healthy human volunteers. It was concluded that the ODT formulation containing ammonium glycyrrhizinate was able to mask the bitter taste of the drug [48]. Caffeine citrate orally disintegrating tablet was formulated using hot-melt extrusion technology to mask the unpleasant bitter taste of the drug. The polymer used was ethylcellulose, along with a suitable plasticizer. Mannitol and crospovidone were also used in the formulations. Both an e-tongue and human gustatory evaluation were used to assess taste masking. The results indicated that the formulations significantly masked the unpleasant taste of caffeine citrate [49].

Cyclodextrins and ion exchange resins have also been studied for their ability to taste-mask cetirizine HCl when formulated in a freeze-dried oral formulation. A pre-formed resinate of cetirizine HCl and various cyclodextrins was incorporated into the Zydis^®^ (Catalent Pharma Solutions, Baltimore, MD, USA) oral lyophilizate. In studies which included a human taste trial, 80% of volunteers reported that the formulation that was taste masked using beta-cyclodextrin and cherry/sucralose flavor system was acceptable [50]. Taste masking of the extremely bitter and metallic-tasting drug phencynonate hydrochloride was studied using novel ion-exchange resins. Drug–resin complexes were prepared and characterized, following which the taste-masking effect was evaluated in healthy volunteers of ODTs prepared using the complexes. ODTs with a drug–resin ratio of 1:1 was found to have a low bitterness index [51].

A study combining the use of a new disintegration testing equipment OD-mate and the SA501C taste-sensing system was capable of predicting the palatabilities, disintegration properties and bitterness intensity of famotidine orally disintegrating tablets [52].

Taste masking and formulation of ranitidine ODT was studied. The ODT formulation of ranitidine containing sodium CMC (carboxymethyl cellulose) was found to have enhanced taste masking potential [53]. Olopatadine hydrochloride, which is a second-generation antihistamine and is widely used for treating allergic rhinitis, was formulated as an ODT. Citric acid was used to taste mask this ODT formulation of olopatadine and it was concluded from gustatory sensation tests that citric acid could suppress the bitterness of olopatadine ODTs in a dose-dependent manner. ODTs containing a combination of 2.5% citric acid, yogurt flavoring, and aspartame exhibited low bitterness and acceptable palatability [54].

Using a novel approach, researchers formulated an orally disintegrating tablet containing 60% of metformin-acesulfame salt prepared by direct compaction and an anion exchange reaction. This modified “sweet” salt demonstrated remarkable improvement of taste and significant enhancement in tablet ability compared to the commercial hydrochloride salt and was caused by the different crystal structures [55].

## 5. Desired Physical Attributes of ODTs

Important parameters to be considered during product formulation design of ODTs include size of the tablet, weight of the tablet, and solubility of components [1]. Other parameters that are routinely studied include disintegration time, robustness as determined by tablet friability and crushing strength, tablet wetting time and physical and chemical stability of the overall formulation. Palatability is a major factor to be considered especially when the ODT is to be used in pediatric patients. Pediatric patients’ compliance is influenced by taste and texture. Important attention needs to be paid to the quantities and types of flavoring and sweetening agents used especially if the ODT is intended for repeated administration [56]. It was demonstrated by the formulation of acetaminophen as ODTs, wherein taste-masked particles of acetaminophen were prepared using tetraglycerol polyricinoleate and Eudragit E100 and then incorporated into ODTs, that use of the disintegrant crospovidone exhibited the shortest disintegration time as well as suppressed drug release in a pH 6.8 test solution and rapid release into a pH 1.2 test solution, which as per the authors, makes it useful as an ODT that suppresses dissolution of acetaminophen in the oral cavity [57]. Some researchers have attempted to address ODT formulation of poorly soluble drugs with poor flowability, by direct compression and with desired release properties. The researchers used Carbamazepine (CBZ) and hydroxypropyl-β-cyclodextrin (HPβCD), respectively, as a model of a poorly soluble drug with poor flowability and as a solubilizing agent. Additionally, Prosolv^®^ (JRS Pharma, Rosenberg, Germany).ODT G2 and F-Melt^®^ (Fuji Chemical Industries Co.,Ltd.,Toyama, Japan) type C were used as the five-in-one co-processed excipients. It was reported that the simultaneous use of co-processing and cyclodextrin technologies rendered ODTs with an in vitro disintegration time in accordance with the European Pharmacopoeia requirements and with a fast and complete drug dissolution. Also, the authors concluded that ODTs containing CBZ/HPβCD (carbamazepine hydroxypropyl beta cyclodextrin) complex could be prepared by direct compression through the addition of co-processed excipients [58]. Some other novel approaches include ODTs with multi-channel structure designed to provide a rapid disintegration and subsequently drug dissolution. The ODTs were prepared using conventional wet compression through perforating channels with a special multi-channel mold. This study demonstrated that the presence of channels could accelerate the disintegration of ODTs because the channels could shorten the distance of water penetration and increased the specific surface area, resulting in a significant reduction in disintegration time. Above all, the introduction of novel multi-channel ODTs provided an alternative preparation method for ODTs and achieved good disintegration characteristics [59].

There have been recent developments in modifying and enhancing testing of physical attributes of ODTs. Researchers conducted a study to develop an in vivo relevant ODT disintegration test named as the Aston test to better represent the oral cavity environment and included lower volume of disintegration medium and relevant temperature and humidity conditions. Furthermore, the Aston test was able to differentiate between different ODTs with small disintegration time windows, as well as between immediate release tablets and ODTs. As per the researchers, this newly developed Aston test provided higher correlations between ODT properties and disintegration time compared to the USP test method and resulted in a linear in vitro/in vivo correlation compared with a “hockey stick” profile of the USP test [60].

## 6. Perspectives on Current and Future Challenges and Opportunities with ODT Dosage Forms

As described in the previous sections, ODTs are a dosage form that can achieve high levels of patient compliance. Consequently, the formulation of ODT dosage form remains an area of great interest among formulation scientists. Table 1 lists challenges, clinical opportunities, and the future potential of ODT dosage forms.

## 7. Conclusions

With the evolution of personalized medicine and its importance in pharmacotherapeutics, the use of ODTs in pediatric and geriatric patients and for certain disease states will be a major area of pharmaceutical product development. Newer technology, such as 3D printing, that can be used in the preparation of these ODTs will aid in the preparation of highly customized and personalized dosage forms. Single or combination ODTs will also be able to meet the needs of patients both medically and financially.

Not many biologics have been subject to ODT formulation technology for obvious reasons of drug degradation by first pass metabolism. However, drugs with a wide variety of therapeutic indications such as proton pump inhibitors, NSAIDs (Non-Steroidal Anti-Inflammatory Drugs), antipsychotics, antiemetics, antihistamines etc., have been successfully formulated as ODTs.

## Figures and Tables

**Table 1:** Challenges, clinical opportunities, and future of ODTs.

Challenges	Clinical Opportunities	Future of ODTs
Limited or very low aqueous solubility of new drugs or APIs (active pharmaceutical ingredients) may pose significant challenges when formulated as ODTs	ODTs can be formulated for marketed drugs which currently do not have a commercially available ODT dosage form (especially for pediatric and geriatric patients)	3D printing technology advancements will advance the creation of personalized or patient specific ODTs
ODTs of complex biological drugs (proteins, peptides etc.) because of their inherent unstable nature and degradation in the harsh environment of the GI tract would need to be overcome by developing ODT matrices that protect the drug	Rapid onset of action of ODT dosage forms make them ideal for acute and chronic pain purposes	Controlled or sustained release ODTs present unique opportunities for expanding the therapeutic benefits offered by ODTs
	Drug combination ODTs (bilayer ODTs) can result in financial savings for patients and also have the benefit of serving as personalized medicine	Research investigating (a) the use of novel materials for taste masking & (b) more relevant testing of ODT disintegration times & disintegration media will continue to evolve
